# Hepatic and immune modulatory effectiveness of lactoferrin loaded Selenium nanoparticles on bleomycin induced hepatic injury

**DOI:** 10.1038/s41598-024-70894-6

**Published:** 2024-09-10

**Authors:** Khaled G. Abdel-Wahhab, Mahmoud Ashry, Laila K. Hassan, Marwa H. El-Azma, Ghada M. Elqattan, Mohamed H. A. Gadelmawla, Fathia A. Mannaa

**Affiliations:** 1 Medical Physiology Department, National Research Centre, Giza, 12622 Egypt; 2 Zoology Department, Faculty of Science, Al-Azhar University, Assuit, Egypt; 3 Dairy Department, National Research Centre, Giza, 12622 Egypt; 4 Histology Department, Faculty of Dentistry, Sinai University, Kantara, Egypt

**Keywords:** Nano-Selenium, Bleomycin, Liver, Lactoferrin, Fibrosis, Rats, Biochemistry, Biotechnology, Chemical biology, Physiology, Health care, Medical research, Molecular medicine

## Abstract

This study aimed to estimate the hepatic and immune ameliorating potential of extracted bovine lactoferrin (LF), Selenium nanoparticles (SeNPs) or their combination (LF/SeNPs) against bleomycin (BLM) induced hepatic injury. Fifty adult male rats (160–200 g) were equally divided into five groups: (1) the saline control group, (2) BLM-injected (15 mg/kg twice a week, ip), and (3–5) groups treated orally with LF (200 mg/kg/day), SeNPs (0.0486 mg/kg/day) or LF/SeNPs combination (200.0486 mg/kg/day) for 6 weeks post BLM-intoxication. Blood and liver samples were subjected to biochemical, histopathological, and immunohistochemical analyses. The results revealed that BLM caused a significant increase in hepatic lipid peroxidation and nitric oxide, as well as serum markers of liver functions (AST, ALT and GGT activities), and levels of GM-CSF, CD4, TNF-α, IL-1β, TGF-β1, fibronectin, triglycerides, cholesterol and LDL-C. Additionally, hepatic glutathione, Na^+^/K^+^-ATPase, and glutathione peroxidase, as well as serum HDL-C, total protein and albumin levels were significantly reduced. Moreover, BLM injection resulted in marked histopathological alterations and severe expression of caspase 3. Post-treatment of BLM-intoxicated rats with LF, SeNPs or LF/SeNPs combination obviously improved the BLM-induced hepatic damages; this was achieved from the marked modulations in the mentioned parameters, besides improving the histopathological hepatic architecture. It is worth mentioning that LF/SeNPs exerted the greatest potency. In conclusion, the obtained results demonstrated that LF, SeNPs and LF/SeNPs succeeded in attenuating the BLM-induced hepatic dysfunction. Therefore, these supplements might be used to protect against drug-associated side effects.

## Introduction

Bleomycin (BLM), an antibiotic, is a glycopeptide isolated from the bacterium, *Streptomyces verticillus*. BLM is used as an antineoplastic drug in the treatment of lymphomas, testicular carcinomas, germ cell tumors, Kaposi’s sarcomas and squamous cell carcinomas^1^. Despite its benefits in the treatment of human cancers, BLM is largely restricted because it has many acute and chronic side effects such as hepatotoxicity and pulmonary toxicity, which results in the formation of fibrogenesis^2^. BLM can induce production of reactive oxygen species (ROS) and cleaving in DNA strands^1^. However, increased ROS production which is involved in the development of fibrosis can be the cause of many serious side effects and may lead to serious or even fatal organ dysfunction^3^. It is clearly that the toxicity of BLM is mainly associated with oxidative stress. Thus, combined therapy with nutrients which have antioxidant ability may be effective for the protection.

Selenium (Se), a micronutrient, is required for good physiological function^4^. Selenium is an important trace element essential for the normal growth and health of both humans and animals^5^, as it participates in important biochemical reactions in the body and in the structure of many selenoenzymes such as glutathione reductase and glutathione peroxidase that perform important roles in maintaining antioxidant pathways, improving the endocrine and immune system, muscle function, reproduction, and tumor prevention^4^. Many studies have demonstrated that Se can prevent oxidative stress and apoptosis caused by heavy metals such as arsenic, nickel, chromium, and cadmium^6^. Selenium has dual effects, as the high Se levels can cause toxic symptoms such as fatigue, dizziness, irritability, gastrointestinal disturbance, nausea and hair loss^7^, while its deficiency also causes health problems such as muscular dystrophy, cardiomyopathy and chronic degenerative diseases^4^. Due to the toxicity and narrow therapeutic margin of traditional Selenium, it was necessary to use Selenium nanoparticles (SeNPs) as a modern approach to solve the problem of malabsorption and toxicity of selenite. Several studies demonstrated that SeNPs have better bioavailability, higher biological activity and lower toxicity than organic and inorganic selenium compounds^8^. Furthermore, the use of SeNPs as chemo preventive and anticancer agents is also well certified^9^.

Lactoferrin (LF), an iron-binding glycoprotein; it is a portion of the transferrin proteins and is found in high concentrations in the milk of mammals and bovine^10^. It is a siderophilic protein with 2 iron-binding sites and has a multitude of physiological functions including iron-chelation, anti-viral, anti-inflammation, anticancer, as well as immunomodulatory-enhancing effects^11^. These functions depend closely on the structural integrity and the high conformational order of the LF. It is mainly extracted from cow's milk and is later added to many commercial products such as nutritional supplements^10^. The antioxidant effects of LF have been attributed to its ability to bind iron ions which is due to the presence of two globular subunits, each subunit can bind a single ferric ion tightly but reversibly^12^. Additionally, LF binding property involves also simultaneous binding of divalent ions such as CO_3_^2−^, Ca^2+^, Zn^2+^ and Cu^2+^^13^. Thus, the purpose of the present study was to compare the hepatoprotective potential of the treatment of Selenium nanoparticles and lactoferrin and to know if their combination exerts synergistic ameliorative action against bleomycin-induced liver damage in animal model.

## Material and methods

### Chemicals

Bleocel (Bleomycin, 15 units vials of lyophilized powder) was purchased from CELON LABS Company, Telangana, India. Selenium nanoparticles (150 nm particle size, 0.15 wt.% water dispersion, CAS number: 7782-49-2) were purchased from Sigma-Aldrich.

### Extraction of lactoferrin

Bovine LF is a glycoprotein with a non-heme bound iron with isoelectric point of 9.5, and highly cationic property which is one of the major important characteristics of LF structure, and responsible for its significant biological features^14^. Fresh bovine raw milk was obtained from Animal Production Research Institute, Agriculture Research Centre, Giza, Egypt. Bovine whey was prepared from bovine raw milk as previously described^15^ with slight modification. The obtained clear LF fractions were tested for their purity and molecular weight using 12% SDS-PAGE^16^. The pure LF was preserved at − 20 °C until further application.

### Lactoferrin-Selenium nanoparticles (LF/SeNPs) combination

A combination of LF/SeNPs was prepared by dissolving LF (1 g) and SeNPs (1 mg) in 10 ml deionized water, then the solution was cool-stirred (4 °C) for 60 min, then followed by cool-centrifugation at 4500 rpm for 10 min at 4 °C, then the precipitate was washed with phosphate buffer saline and recentrifuged^17^. The un-ligated metal ions, in the precipitated LF/SeNPs combination, were then removed thorough washing by 0.1 M sodium bicarbonate though dialysis. Then, the dialyzed LF/SeNPs were freeze-dried to produce a solid powder before being stored at –80 °C for further applications. Regarding the capacity of lactoferrin to bound Se, Higuchi et al.^17^ reported that each 100 g lactoferrin can bound 24.3 mg of Selenium; therefore, the calculated dose that used in this study was 200.0486 mg of LF/SeNPs combination per kg; this dose is equivalent to a dose of 200 mg LF/kg^18^ or equivalent to dose of 0.0486 mg SeNPs/kg^19^.

### Experimental design

Adult male Wistar albino rats weighing 160–200 g were obtained from Animal Colony, National Research Centre, Giza, Egypt; the animals were housed in plastic cages and fed with standard laboratory diet and water ad libitum. All animals received human care in accordance with the institutional standards for the handling and use of experimental animals, where the study proposal was approved by the ethical committee of faculty of science, Al-Azhar university, Assuit (approval No AZHAR 14/2023). All methods were carried out in accordance with ARRIVE guidelines.

After acclimation for a week, all animals were randomly divided into five groups (10 rats each) as follows: the first group was injected intraperitoneally with saline and served as control; the second group was injected intraperitoneally, twice a week, with 15 mg/kg of BLM dissolved in sterile saline for six weeks^20^; the third and fourth groups were injected intraperitoneally with BLM for 6 weeks, then orally administrated with either LF (200 mg/kg/day)^18^ or SeNPs (0.0486 mg/kg/day)^19^ for other 6 weeks; the fifth group was intoxicated with BLM for 6 weeks, then treated with LF/SeNPs formulation (200.0486 mg/kg/day that included 200 mg LF and 0.0486 mg SeNPs) for other 6 weeks.

### Blood and tissue sampling

At the end of the treatments period, rats were fasted overnight and following anesthesia by isoflurane inhalation, blood samples were withdrawn from the retro-orbital plexus using heparinized-sterile glass capillaries. The coagulated blood samples were cool centrifuged at 3000 rpm for 15 min and the sera were separated and stored at − 80 °C till biochemical analysis. After blood collection, the animals were killed by sudden decapitation, and then one part the liver of each animal was dissected out, washed in saline, dried, rolled in a piece of aluminum foil, and stored at − 80 °C for determination of oxidative stress markers until homogenization could be completed. Hepatic tissue was homogenized in ice-cold phosphate buffer (50 mM, pH 7.4) to give 10% homogenate (w/v); the homogenate was cool centrifuged at 7000 rpm for 20 min to remove the nuclear and mitochondrial fractions; finally, the supernatant was divided into aliquots and stored at − 80 °C until the determination of Na^+^/K^+^ ATPase activity and oxidative stress markers. Another part of the liver was soaked in formalin-saline (10% v/v) buffer for histopathological examinations.

### Determinations of liver functions and lipid profile

The activity of serum alanine aminotransferase (ALT), aspartate aminotransferase (AST), alkaline phosphatase (ALP) and gamma-glutamyl transferase (GGT), as well as levels of toal protein and albumin were detrmined spectrophotometrically using reagent kits purchased from CliniSciences, France. Total cholesterol, triglycerides, LDL-cholesterol (LDL-C) and HDL-cholesterol (HDL-C) levels were tested using reagent kits purchased from Cell Biolabs, USA.

### Oxidative stress markers and Na^+^/K^+^-ATPase activity

Lipid peroxidation (measured as malondialdehyde, MDA) level in liver homogenate was estimated chemically according to the method described by Ruiz-Larrea et al.^21^ on the base of MDA reaction with thiobarbituric acid which forms a pink complex that can be measured photometrically; while nitric oxide (NO) and reduced glutathione (GSH) levels as well as the enzymatic activity of glutathione peroxidase (GPx) were determined spectrophotometrically using kits obtained from Biodiagnostic Co., Giza, Egypt. Sodium–potassium–adenosine triphosphatase (Na^+^/K^+^-ATPase) activity was determined according to the modified chemical method that described by Tsakiris et al.^22^.

### Estimation of inflammatory and profibrotic markers

CD4 level was measured using rat ELISA reagent kits purchased from Sunlog Biotech, Zhejiang, China. Serum levels of granulocyte–macrophage colony-stimulating factor (GM-CSF), interleukin-1beta (IL-1β), tumor necrosis factor-alpha (TNF-α) and transforming growth factor-beta (TGF-β) were determined using rats’ reagent ELISA kits purchased from SinoGeneClon Biotech Co., Hang Zhou, China. Fibronectin level was measured using rat ELISA reagent kits purchased from Sunlog Biotech, Zhejiang, Chia.

### Histopathological analysis

The excised liver tissues of all rats were fixed for 24 h in 10% neutral buffered formalin, washed in running water, dehydrated with ethanol, followed by xylene for clearing, paraffin embedding, and sectioning, then the sections were stained with Hematoxylin and Eosin stains for evaluation by the light microscope^23^.

### Immunohistochemistry (IHC) analysis

Five µm thickness sections of the processed liver tissues from each rat for all groups were immune-stained for 90 min with anti-Caspase-3 primary antibody. Following the application of the secondary antibody via the immunoperoxidase method, the sections were stained using the Universal Staining Kit, "DAB."(ScyTek Laboratories, Inc. Logan, UT, USA) at room temperature for half an hour^24^.

### Quantitative evaluation of IHC staining

Semi-quantitative IHC is a powerful method for investigating protein expression and localization within tissues. The semi-quantitative IHC involves using ImageJ Fiji software (Johannes Schindelin, Albert Cardona, Mark Longair, Benjamin Schmid, and others, https://imagej.net/Fiji/Downloads), version 1.2 (no specific plugin was used) to conduct deconvolution and downstream analysis. The area percent for positive caspase-3 immunoreaction was measured at magnification X 400 for all groups^25^.

### Statistical analysis

Comparisons between means were carried out using one-way analysis of variance (ANOVA) followed by post hock (Tukey) multiple comparisons test at p ≥ 0.05. This was carried out using statistical analysis system (SAS) program software; copyright (c) 1998 by SAS Institute Inc., Cary, NC, USA.

### Ethics declarations

All animals received human care in accordance with the institutional standards for the handling and use of experimental animals, where the study proposal was approved by the ethical committee of faculty of science, Al-Azhar university, Assuit (approval No AZHAR 14/2023). All methods were carried out in accordance with ARRIVE guidelines.

## Results

### Extraction and characterization of lactoferrin

**Fig. 1:**
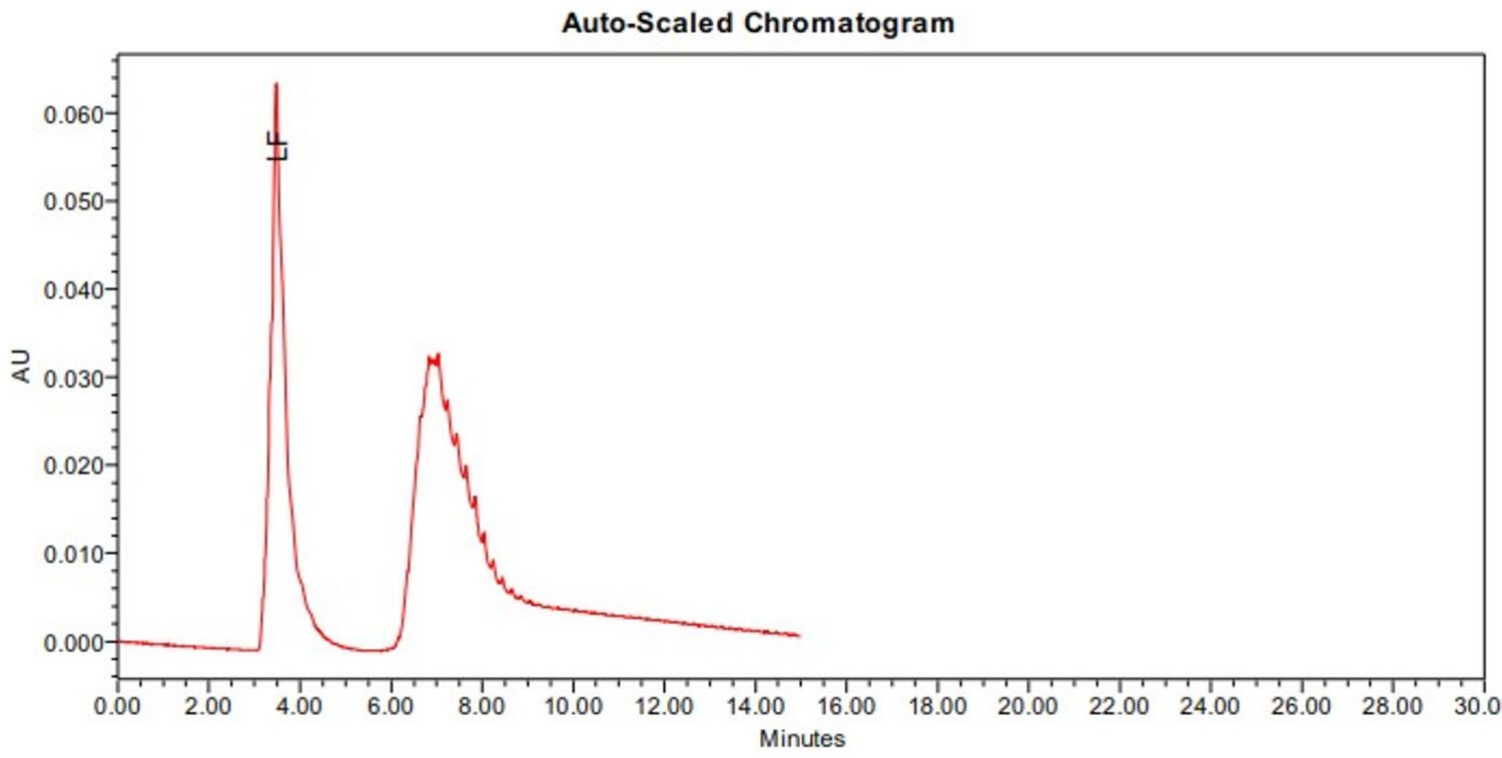
Chromatogram of the purified lactoferrin extracted from raw bovine milk.

The extraction, isolation, and purification process of lactoferrin revealed that bovine milk contains 3.92 mg/ml. The HPLC characterization of the pure-isolated lactoferrin is illustrated in Fig. 1.

### Biochemical results

In the present study, BLM-intoxication resulted in a significant elevation in the activities of serum ALT, AST, ALP and GGT coupled with a significant decrease in levels of albumin and total protein as compared to the control group. In a favorable manner, post-treatment of BLM-intoxicated animals with either lactoferrin or SeNPs showed a significant improvement in the mentioned parameters although their values were still away from those of the control group. Moreover, animals intoxicated with BLM and having received the LF/SeNPs recorded values comparable to those of the control (Table 1).

**Table 1:** Liver function tests of the studied groups.

	Control	BLM	BLM/LF	BLM/SeNPs	BLM/LF/SeNPs
ALT (U/L)	74.40 ± 6.67	135 ± 11.53*	89.19 ± 7.09^#^	93.79 ± 6.44^#^	82.69 ± 4.74^#^
AST (U/L)	112 ± 15.03	240 ± 3.86*	158 ± 11.62^#^	149 ± 13.26^#^	127 ± 13.86^#^
GGT (U/L)	24.72 ± 1.50	51.70 ± 2.20*	36.30 ± 3.11^#^	34.05 ± 4.29^#^	29.46 ± 4.02^#^
ALP (U/L)	8.06 ± 0.32	24.06 ± 1.65*	16.00 ± 1.31^#^	15.40 ± 1.61^#^	11.04 ± 1.67^#^
Protein (g/dl)	7.97 ± 0.13	4.08 ± 0.21*	5.55 ± 0.11^#^	5.72 ± 0.16^#^	6.25 ± 0.07^#^
Albumin (g/dl)	4.91 ± 0.11	2.70 ± 0.05*	3.26 ± 0.06^#^	3.11 ± 0.06^#^	3.51 ± 0.08^#^

Data are presented as mean ± standard error. * is significant versus the control group, while ^#^ is significant versus BLM group at p ≤ 0.05. LF (lactoferrin); BLM (bleomycin); SeNPs (Selenium nanoparticles).

Post-BLM-intoxication, the animals showed significant increases in serum cholesterol, triglycerides, and LDL-c levels, matched with a marked drop in HDL-c level in compared to the control group. Treatment of BLM-intoxicated animals with either LF, SeNPs or LF/SeNPs formulation significantly reversed the levels of the above parameters of lipid profile toward the corresponding values of the normal controls; Attention showed he drown here, the combined LF/SeNPs was more effective than the single treatment of LF or SeNPs (Table 2).

**Table 2:** Lipid profile of the studied groups.

	Control	BLM	BLM/LF	BLM/SeNPs	BLM/LF/SeNPs
Chol (mg/dl)	66.96 ± 1.55	162 ± 4.63*	93.21 ± 5.90^#^	100 ± 5.22^#^	84.07 ± 2.75^#^
Trig (mg/dl)	111 ± 3.04	225 ± 6.83*	139 ± 4.03^#^	132 ± 4.43^#^	126 ± 7.23^#^
LDL-C (mg/dl)	23.03 ± 0.52	61.57 ± 2.35*	41.89 ± 2.84^#^	39.68 ± 1.99^#^	31.28 ± 2.26^#^
HDL-C (mg/dl)	46.16 ± 0.73	32.56 ± 1.19*	41.72 ± 0.47^#^	36.99 ± 0.72^#^	43.74 ± 0.61^#^

Data are presented as mean ± standard error. * is significant versus the control group, while ^#^ is significant versus BLM group at p ≤ 0.05. LF (lactoferrin); BLM (bleomycin); SeNPs (Selenium nanoparticles).

The present study revealed that BLM-intoxication induced a meaningful elevation in the hepatic MDA and NO levels, associated with a remarkable reduction in the values of hepatic content of GSH and activities of GPx and ATPase when compared with the control group. On the other hand, post-treatment with LF, SeNPs, or LF/SeNPs formulation succeeded to markedly decrease hepatic MDA and NO levels; moreover, hepatic GSH content as well as GPx and Na^+^/K^+^-ATPase activities showed a significant increase in compared to the corresponding results of the untreated BLM-intoxicated-group. These supplements, either singly or combined, exhibited an antioxidant-ameliorating potency; this exhibition was more pronounced in the group treated with LF/SeNPs formulation (Table 3).

**Table 3:** Oxidative stress markers and Na^+^/K^+^-ATPase activity of the studied groups.

	Control	BLM	BLM/LF	BLM/SeNPs	BLM/LF/SeNPs
NO (nmol/g)	7.58 ± 0.24	14.88 ± 0.22*	12.26 ± 0.31^#^	11.78 ± 0.37^#^	9.88 ± 0.06^#^
MDA (µmol/g)	623 ± 22.3	1134 ± 30.05*	792 ± 20.4^#^	979 ± 32.8^#^	712 ± 20.3^#^
GSH (µmol/g)	1564 ± 87	823.88 ± 16.7*	1200 ± 25.63^#^	1254 ± 18.94^#^	1340 ± 32.23^#^
GPx (U/g)	8976 ± 93.3	2992 ± 26.7*	4082 ± 50.4^#^	3461 ± 99.6^#^	7220 ± 42.6^#^
Na^+^/K^+^-ATPase (µmol pi/hr/g)	3777 ± 88.31	2199 ± 39.5*	2230 ± 22.14^#^	3396 ± 53.5^#^	2637 ± 54.8^#^

Data are presented as mean ± standard error. * is significant versus the control group, while ^#^ is significant versus BLM group at p ≤ 0.05. LF (lactoferrin); BLM (bleomycin); SeNPs (Selenium nanoparticles).

**Fig. 2:**
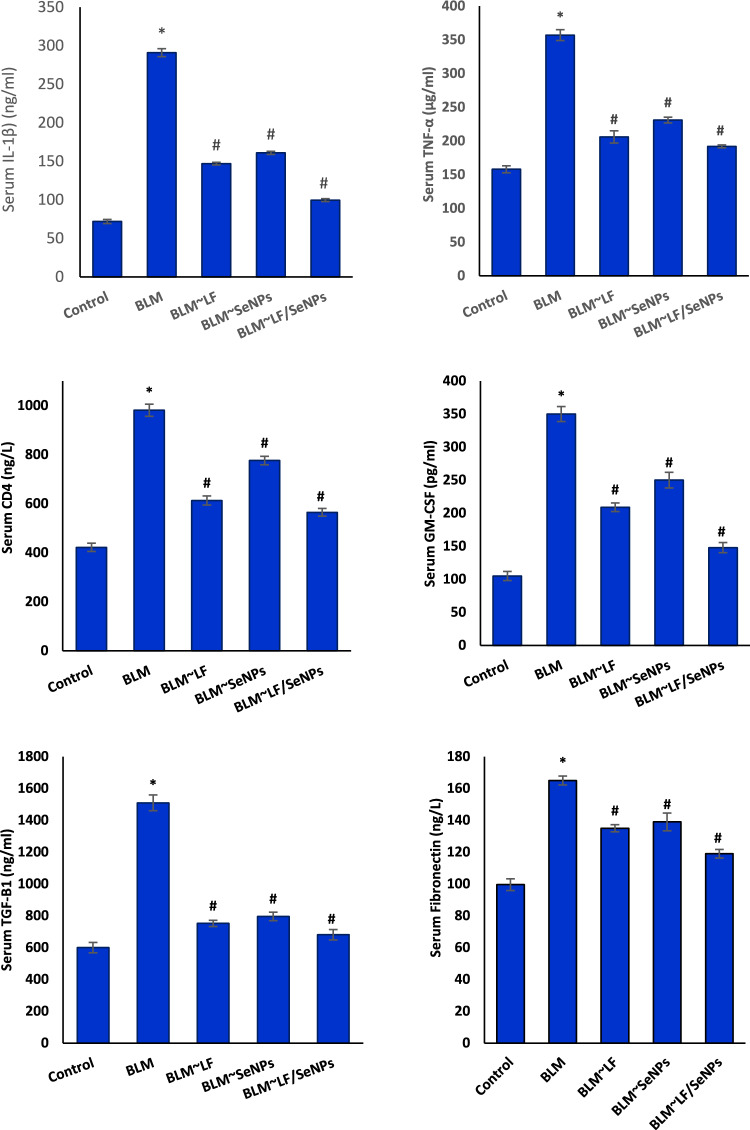
Effect of different treatments on serum CD4, GM-CSF, TNF-α, IL-1β, TGF-β1, and fibronectin levels of BLM-intoxicated rats. * Is significant versus the control group, while # is significant versus BLM group at p ≤ 0.05. LF (lactoferrin); BLM (bleomycin); SeNPs (Selenium nanoparticles).

The obtained data revealed that animals intoxicated with BLM showed significant increases in the serum level of apoptotic markers (GM-CSF and CD4), pro-inflammatory cytokines (TNF-α and IL-1β), and the pro-fibrotic markers (TGF-β1 and fibronectin) in compared to their corresponding values of control group. Favorably, treatment of BLM-intoxicated animals with LF, SeNPs, or LF/SeNPs formulation showed a significant amelioration in the values of all above-mentioned markers. It is worth noting that treatment with LF/SeNPs formulation exerted the most remarkable improvement (Fig. 2).

### Immunohistochemistry analysis

**Fig. 3:**
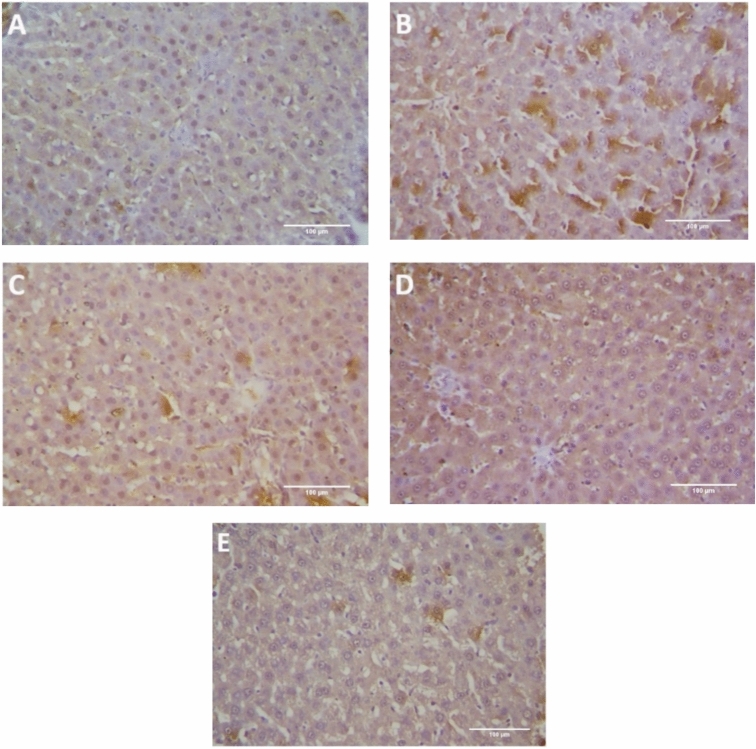
Photomicrographs of liver tissues of the different study groups. The anti-caspase-3 immunoreactivity represented in the tissues as brown color by DAB chromogen. (**A**) The control group showed weak cytoplasmic caspase-3 immunoreactivity; (**B**) The BLM-injected group showed severe caspase-3 immunoreactivity; (**C**) The BLM ~ LF group showed mild caspase-3 immunoreactivity; (**D**) The BLM ~ SeNPs group showed moderate caspase-3 immunoreactivity; (**E**) The BLM ~ LF/SeNPs group showed a weak, approximately normal, caspase-3 positivity.

**Fig. 4:**
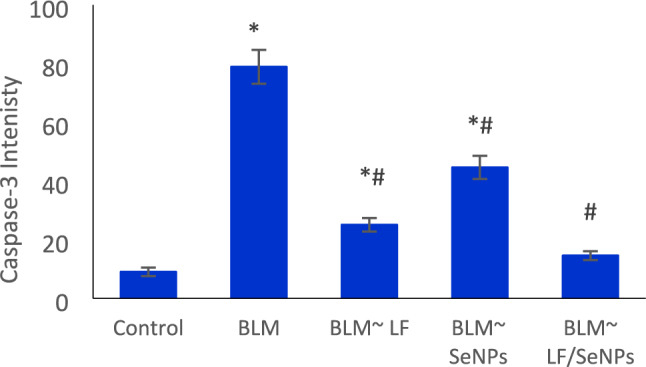
Intensity quantitative measurement for positive caspase-3 immune reactions. Data are presented as mean ± standard error; subjected to one-way ANOVA followed by post hoc (Duncan) test at p ≤ 0.05; * is significant versus the control group, while # is significant versus BLM group; LF (lactoferrin); BLM (bleomycin); SeNPs (Selenium nanoparticles).

The immunohistochemistry analysis revealed the occurrence of a very weak expression of caspase-3 in liver tissue of the control rats (Fig. 3A). The BLM-intoxication group showed severe expression of caspase-3 (Fig. 3B). The other groups revealed a reduced caspase-3 immunoreactivity in compared to the untreated BLM-intoxicated group (Fig. 3C–E), with approximately normal caspase-3 expression in LF/SeNPs formulation treated-group (Fig. 3E). There was a significant difference between BLM-intoxicated group and the groups treated with LF, SeNPs or LF/SeNPs formulation regarding caspase-3 IHC immunoreactivity with substantial reduced caspase-3 positivity in the treatment groups (Fig. 4).

### Histopathological examination

**Fig. 5:**
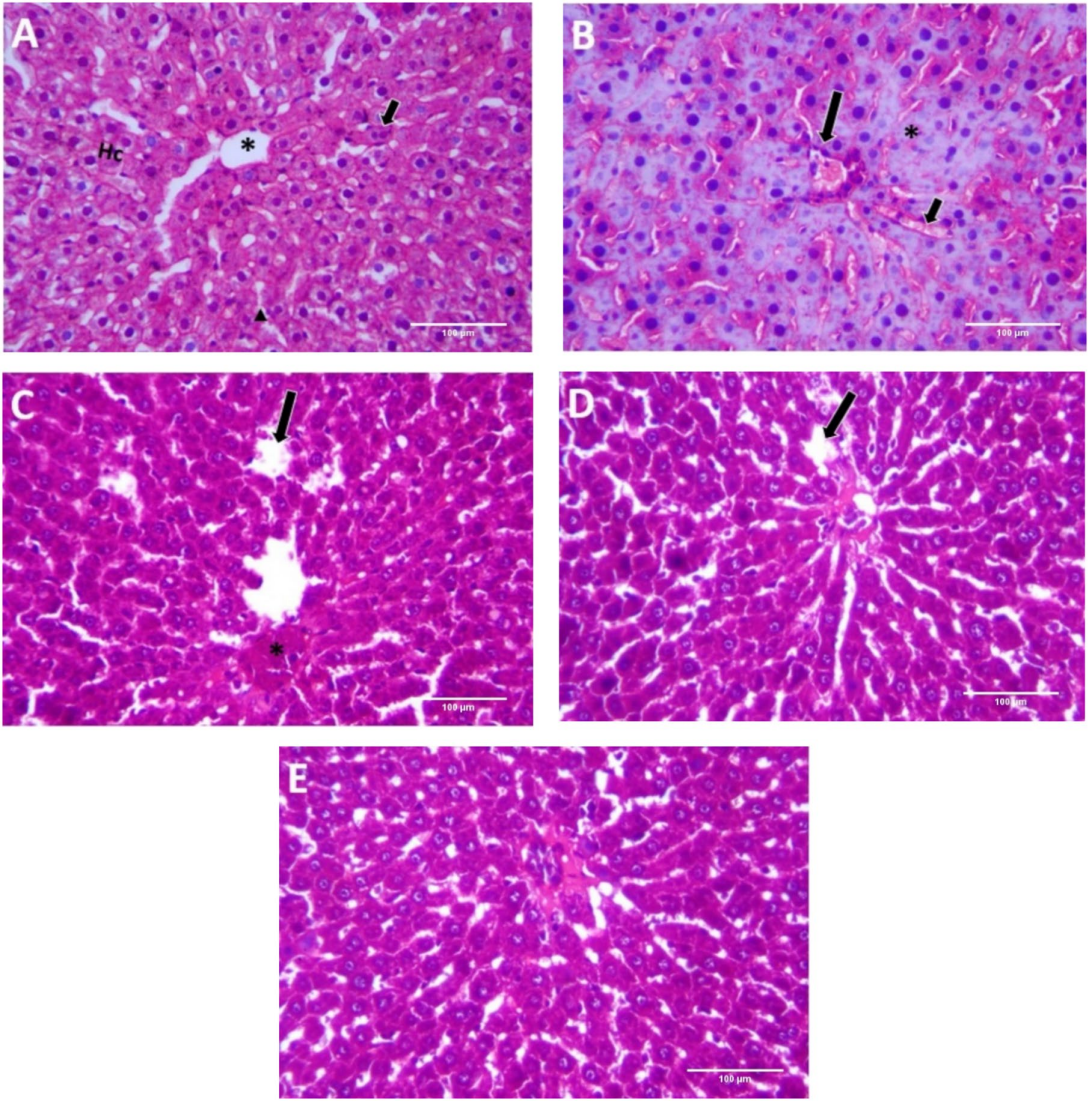
Photomicrographs of liver tissues of the different study groups; (**A**) Control group showing normal hepatic lobule structure with central vein (*), hepatic cord (Hc), binucleated cell (short arrow) and hepatic sinusoids with epithelial cells (arrowhead); (**B**) BLM-intoxicated group showed focal necrosis with pycnotic nuclei (*), congested bile duct (long arrow) and obliterated sinusoids (short arrow); (**C**) BLM ~ LF treated group showed focal necrosis (*), and congested sinusoids (long arrow); (**D**) BLM ~ SeNPs treated group showed congested sinusoids (long arrow) with normal hepatic pattern; (E) BLM ~ LF/SeNPs formulation treated group showed approximately normal architecture.

The histopathological examination of the control group liver tissues showed normal structure of hepatic lobules with branching, radiating, and anastomosing hepatic cords with the central vein. The hepatic sinusoids in between the hepatic cords were lined with squamous endothelial cells with flat nuclei. The hepatocytes were polygonal in shape with central rounded vesicular nuclei and acidophilic cytoplasm with few binucleated hepatocytes (Fig. 5A). BLM-treated group revealed marked histopathological alterations. Most of the hepatocytes displayed marked necrosis with remarkable pyknotic nuclei (Fig. 5B). BLM /LF and BLM /Selenium NPs-treated groups showed normal hepatic structures with some congested hepatic sinusoids and focal necrosis in BLM /LF group (Fig. 5C, D). The BLM /LF/Selenium NPs-treated group showed improved histological structure of hepatic lobules and rearranged radiating hepatic ducts (Fig. 5E).

## Discussion

The liver has a vital role in metabolism and detoxification of xenobiotics, metabolites, and drugs^26^. Bleomycin, an anticancer drug, is inactivated by bleomycin hydrolase in hepatic cells. With the deficiency of BLM hydrolase in liver, BLM exerts its toxic damage through the induction of oxidative stress and DNA damage^27^. BLM can damage DNA through the formation of DNA/Fe2+/BLM complex which leads to the generation of ROS, including superoxide and hydroxyl radicals that damage DNA, lipids, and proteins^28^. Oxidative stress occurs when the capability of the anti-oxidative defense system in eliminating the free radicals and ROS decreases; oxidative damage occurs because of increased lipid peroxidation and diminution of the antioxidant effects of thiol groups in organs^29^.

In this study, hepatocytes damage is evidenced by the marked increments in the values of serum ALT, AST, ALP and GGT and the decrement in levels of albumin and total protein in BLM-treated group. Similar observations were recorded by Kandhare et al.^28^. These markers are considered as indicators that confirm the presence of liver dysfunction. In the case of hepatic membrane damage, liver enzymes are released into the blood and are measured in blood serum as markers of liver injury^30^. Additionally, lipid profile was disturbed also by BLM treatment. Lipids concentration is correlated with the metabolic functions which are affected by liver integrity. Lipids like cholesterol and triglycerides were found to increase in various hepatic diseases. Also, Seki et al.^31^ reported that lipid content in liver cells is affected by the integrated activities of cellular enzymes that catalyze lipids metabolism.

In the present study, oxidative injury in BLM-treated rats appeared from the marked reduction in GSH and GPx values. GSH is an antioxidant tripeptide, it improves protection against oxidative agents-induced cell damage^32^. GPx which is a Seleno-proteins enzyme; it functions as protector and detoxifier from oxidative injury via free radicals scavenging^33^. Oxidative damage was asserted also by increased MDA and NO content, and histological changes in liver tissue. MDA is the end aldehyde product of peroxidation of the polyunsaturated fatty acids of cell membrane^34^. Our results are consistent with other studies, which demonstrated the involvement of lipid peroxidation and oxidative stress in BLM ‑induced liver injury^2^. An increase in NO levels has been reported also because of BLM-induced increases in inducible nitric oxide synthase (iNOS) at gen and protein levels^35^. The overproduction of NO by iNOS plays a vital role in inducing various inflammatory diseases including liver fibrosis^36^ as NO reacts with superoxide radical and forms highly reactive peroxynitrites which lead to tissue injury^37^.

Inflammatory mediators have been reported to be involved in BLM toxicity^38^. Elevated levels of GM-CSF and CD4 were observed in serum of bleomycin group**.** GM-CSF is a monomeric glycoprotein, functions as a cytokine, secreted by activated T cells, natural killer cells, and mast cells. GM-CSF stimulates stem cells to produce dendritic cells and macrophages^39^ and stimulates also macrophages and monocytes to secrete pro-inflammatory cytokines^40^. CD4, a monomeric glycoprotein, is produced also by blood T lymphocytes; it has been suggested that CD4 participates in the signal transduction during T cell activation^41^. It has been reported that the CD4 T lymphocyte can induce some degree of fibrotic response; in this concern, Piguet^42^ reported that the depletion of CD4 T lymphocytes by treatment with the anti CD4 mAb restricted the bleomycin-induced elevation of TNF mRNA level and fibrosis.

Serum levels of TNF-α, IL-1β and TGF-β1 in the current study exhibited a marked increase in BLM –treated animals. The release of these cytokines appears to be the main mediators of hepatic fibrosis^43^, Fibronectin also increased by BLM treatment. BLM has been reported to increase the release of the pro-inflammatory cytokines (TNF-α and IL-1β) via elevating ROS through the formation of the DNA/Fe2+/BLM complex^38^. These sinister events cause increased expression of TGF-β1, and activation of hepatic stellate cells that ultimately leads to the synthesis of scar-forming collagen, and consequently the development of liver fibrosis^28^. TGF-β1 is a pro-fibrotic cytokine that can directly stimulate the differentiation of fibroblasts into myofibroblasts^44^, through stimulation of overexpression of fibronectin and collagen which are the main proteins of extra cellular matrix involved in the fibrotic process^45^. Although there is an elevation in the level of fibronectin in the BLM group in our study, this increment did not enough to develop liver fibrosis. This may be due to the short duration of BLM treatment. These results are asserted by the results of Vásquez-Garzón et al.^2^ who reported that the fibrotic process in the liver takes place similarly to that has been occurred in the lung, but to a lesser extent due to the presence of hepatic bleomycin hydrolase.

The inflammatory effects have been reported to increase expression of the pro-apoptotic markers^46^. The immunohistochemistry analysis revealed the occurrence of severe expression of the apoptotic marker, caspase-3, in liver cells of BLM-treated group. This marker is markedly stimulated in the cells that are exposed to cytotoxic agents and it plays a vital role in tumorigenesis after cell injury^47^.

Na^+^/K^+^-ATPase is an enzyme found in the plasma membrane in all animals^48^. It controls the active transport of potassium and sodium across the cell membrane; it also controls cell volume, muscle, and nerve signals, and boosts the transport of amino acids and sugars across the cell membrane. Na^+^/K^+^-ATPase is highly lipid-dependent; therefore, peroxidation of membrane lipids affects this enzyme; Bleomycin hepatotoxicity therefore leads to membrane lipid peroxidation that causes disturbance in membranes lipid structure, resulting in inhibiting Na^+^/K^+^-ATPase activity that was observed in the current study. Li et al.^49^ found a significant negative correlation between the Na^+^/K^+^-ATPase and profibrotic molecules in alveolar epithelial cells (AECs) of patients with idiopathic pulmonary fibrosis (IPF). The findings of these authors revealed that the downregulation of the Na^+^/K^+^ ATPase β1 subunit enhances the expression of profibrotic protein in patients with IPF and a mouse model of pulmonary fibrosis.

The oxidative damage, caused by BLM, is considered the main mechanism that leads to the subsequent hepatic injury; therefore, the supplementation of natural antioxidants has been proposed to counteract the harmful effects of BLM and prevent liver injury. Administration of lactoferrin, SeNPs or their combination to BLM ‑treated animals was greatly effective in decreasing lipid peroxidation and improving antioxidant capability in the liver of BLM‑treated group. This emerged from the marked decrease in hepatic MDA and NO contents and the significant rise in GSH and GPx values. This improvement was more pronounced in the animals received the combined treatment of lactoferrin and SeNPs, indicating the synergistic effect between lactoferrin and SeNPs. Mostly, the benefit role of both substances might be attributed to the antioxidant effect and their free radical scavenging property. These results were in line with that reported for lactoferrin which was characterized in the current study by the HPLC analysis and confirmed that of earlier study^11^. The obtained results were in line also with that reported for SeNPs^50^. Consequently, the possible antioxidant effect of SeNPs may be attributed mainly to its participation in the Seleno-proteins structure such as selenocysteine which is the active center of GPx enzyme^51^. This enzyme can help in reducing lipid peroxidation, intracellular hydrogen peroxide^52^ and ROS generated by all the cells^53^. On the other hand, lactoferrin can act as an iron scavenger by direct iron-chelating property or by modification of the key iron-binding proteins^10^.

In addition, lactoferrin, SeNPs and their combination could also significantly counteract the values of serum ALT, AST, ALP, GGT, albumin and total protein, suggesting their membrane stabilizing effect that based on their antioxidant activity, which may be responsible for saving the hepatocytes against the oxidative damage. This in turn improves the liver integrity and function and consequently improves Na^+^/K^+^-ATPase activity, protein synthesis and lipid metabolism. This improvement was more pronounced in the group that taken lactoferrin plus SeNPs.

In consistence with our results, Wang et al.^50^ observed the protective effect of SeNPs against nickel-induced developmental hepatic damage in rats. SeNPs have been found to protect the liver from cadmium-induced toxicity by ameliorating markers of liver function and restoring the activity of antioxidant enzymes. Amin et al.^4^ demonstrated the ameliorative effect of SeNPs against oxidative stress mediating liver injuries induced by acetaminophen toxicity through modulating the oxidative stress and decreasing DNA fragmentation. In another study conducted on rats, SeNPs have been found to attenuate the deleterious effects of hypothyroidism on renal and hepatic functions by normalizing the values of AST, ALT, ALP, creatinine, and BUN^54^. Additionally, Rizk et al.^55^ confirmed the hypolipidemic activity of SeNPs as they noted that adding SeNPs to laying hens diet resulted in lowering cholesterol, triglycerides, and LDL-C, and raised HDL-C compared to the control group. These authors attributed this effect to the lipolysis that increased with Se intake. Moreover, Li et al.^56^ indicated that lactoferrin treatment prevented ethanol-induced liver injury and counteracted the values of AST and ALT in mice by modification of hepatic alcohol metabolism and modulating gut microbiota. Furthermore, ingestion of lactoferrin can help in preventing disturbance of hepatic lipid metabolism, which is associated with non-alcoholic steatohepatitis^57^, feeding with high-fat diet and ethanol ingestion^56^.

The obtained data revealed that lactoferrin, SeNPs and their combination succeeded to decrease the levels of inflammatory mediators (GM-CSF, CD4, TNF-α, IL-1β and TGF-β1) to reach near the normal levels and reduced the fibrotic marker, fibronectin, in rats treated with BLM, suggesting the anti-inflammatory activity of these supplements. Moreover, the combined treatment was more effective than the treatment with lactoferrin or SeNPs alone. SeNPs are known to have anti-inflammatory and antioxidant effects^58^. In addition, it was reported that Se deficiency induces inflammation through the activation of NF-κB^59^ which plays critical role in regulating gene expression of iNOS and inflammatory cytokines such as TNF-α, IL-6, and IL-8^60^. Indeed, as indicated previously, administration of SeNPs to nickel-exposed animals showed a significant amelioration of apoptosis and inhibition of cytochrome c, caspase-3 and caspase-9 through activation of PI3K/AKT signaling pathway^50^. SeNPs have been found also to improve cardiac fibrosis through the modulation of oxidative stress status in hypothyroid rats^61^. Additionally, Aoyam et al.^11^ found that lactoferrin treatment reduced the expression the inflammatory cytokine (TNF-a, IL-6, and IL-1b) and fibrotic related molecules (TGF-β, Timp2, and Col1a1) mRNAs in liver via NF-κB inactivation. Thus, the protection, afforded by either lactoferrin and/or SeNPs against BLM-induced hepatotoxicity is presumably due to their ability to suppress oxidative processes induced by this drug. This beneficial effect might be the result of its direct free radical scavenging properties. Moreover, marked amelioration in the histopathological changes have been observed due to their antioxidant capabilities. Vicas et al.^6^ reported that the administration of SeNPs to cadmium ‑intoxicated rats prevented the morphological changes in the livers of treated rats. Also, lactoferrin was found to markedly improve hepatic steatosis in non-alcoholic steatohepatitis modeled rats^11^ and could suppress chemically induced liver fibrosis^55,62^.

## Conclusion

In conclusion, our study showed that lactoferrin, SeNPs and their combination can attenuate the hepatic dysfunction induced by BLM as indicated by enhancing endogenous antioxidant capacity, and normalizing lipid peroxidation reaction and its consequence related biochemical and histological changes. The study showed also that the combination of lactoferrin and SeNPs exerted the more beneficial effect indicating synergistic effect between lactoferrin and SeNPs. The mode of action of these supplements might be through two different mechanisms. SeNPs increase formation of Seleno-enzymes which act as powerful antioxidant and free radicals’ scavengers while lactoferrin exerts its effect through eliminating the iron ions that combine with BLM and form the toxic DNA/Fe2+/BLM complex. Therefore, lactoferrin and SeNPs may be a potential preventive or ameliorative application against the side effects of the anticancer therapy.

## Data Availability

The authors declare that the data supporting the findings of this study are available within the paper.
